# Good Results for PJI Caused by *Corynebacterium* spp. After Algorithmic Surgical Approach and Rifampicin Combination in Implant Retention

**DOI:** 10.1093/ofid/ofaf146

**Published:** 2025-03-10

**Authors:** Ana Trebše, Samo Roškar, Anže Mihelič, Rihard Trebše

**Affiliations:** University Medical Centre Ljubljana, Ljubljana, Slovenia; Valdoltra Orthopaedic Hospital, Ankaran, Slovenia; Faculty of Medicine, University of Ljubljana, Ljubljana, Slovenia; Valdoltra Orthopaedic Hospital, Ankaran, Slovenia; Valdoltra Orthopaedic Hospital, Ankaran, Slovenia; Faculty of Medicine, University of Ljubljana, Ljubljana, Slovenia

**Keywords:** prosthetic joint infections, total joint arthroplasty, total hip arthroplasty, total knee arthroplasty, corynebacterial infections, algorithmic approach

## Abstract

**Background:**

*Corynebacterium* spp. is a rare culprit in periprosthetic joint infections (PJIs), with limited data available on outcomes and appropriate treatment course. The aim of this study was to evaluate the success rate of a clinical cohort of patients with PJI, where *Corynebacterium* spp. was the causative organism (CPJI), treated according to an institutional algorithm based upon European Bone and Joint Infection Society guidelines.

**Methods:**

From the institutional bone infection registry, 44 patients treated for CPJI between 2007 and 2023 were identified. CPJIs were divided into 2 groups according to the isolated microbes: monomicrobial (14 [32%]) and polymicrobial (30 [68%]). Patients were treated with debridement, antibiotics, and implant retention (DAIR; 14 [32%]) or with 1- or 2-stage implant exchange (reimplantation group; 30 [68%]). In 13 (30%) cases, antibiotic combination with rifampicin was used.

**Results:**

Out of 44 patients, 4 required further treatment. In monomicrobial CPJI, the treatment course was successful in all patients, whereas in polymicrobial CPJI it was successful in 87%. Antibiotic combination including rifampicin was used in 4 monomicrobial cases (29%) and 9 polymicrobial (30%) cases. In the polymicrobial group, DAIR was successful in 90% (9/10), while the reimplantation group had an 85% (17/20) success rate.

**Conclusions:**

In contrast with the previously published papers on CPJI, the results in our cohort were good, with the total cure rate being 91%. The cure rate was slightly lower in the polymicrobial group compared with the monomicrobial: 87% and 100%, respectively. Surgical therapy according to the established institutional algorithm resulted in a high success rate.

## Teaser

Following the algorithmic approach, the total cure rate of corynebacterial periprosthetic joint infections was 91%, slightly lower in the polymicrobial compared to the monomicrobial group. When indicated, DAIR procedure appears as an optimal treatment choice, and the antibiotic combination with rifampicin seems to be beneficial when the implant is retained.

Periprosthetic joint infections (PJIs) represent a significant and serious complication of arthroplasty, often requiring intricate therapeutic approaches due to high morbidity and divergent clinical presentation [1, 2]. Up to 1% of all PJIs can be caused by *Corynebacterium* spp. (CPJI) [2], which are considered atypical. There are limited clinical data available regarding characteristics of and treatment options for CPJI [3, 4]. *Corynebacterium* spp. are gram-positive rods and facultative anaerobes comprising >110 known species that inhabit human skin and mucosal surfaces as microbiota [5]. While traditionally regarded as microbiological contaminants, there is emerging evidence suggesting that *Corynebacterium* spp. can be a causative microorganism, leading to infection of various tissues, including device-associated infections such as PJIs [6, 7]. Current data suggest that they are involved in up to 5% of cases of mixed flora PJI and only 2% of monomicrobial PJI, with the most prevalent species including *C. amycolatum, C. aurimucosum, C. jeikeium,* and *C. striatum* [4, 8–10]. The management of PJI is complex, encompassing both surgical and antimicrobial interventions. Accurate identification of the causative microorganism is crucial for antimicrobial treatment due to challenging antimicrobial susceptibility patterns and limited oral antibiotic options [11]. An exact treatment strategy for CPJI remains elusive, and treatment can be challenging due to a limited spectrum of effective antibiotic options [2, 3, 12].

In this study, we aimed to evaluate the burden of CPJI and examine the success rate of treatment in a single tertiary reference center according to the algorithm [13, 14].

## METHODS

### Data Collection and Cohort

This retrospective cohort study was conducted at a Bone Infection Unit of a single specialized orthopedic center. We included all patients who were treated for PJI between January 2007 and December 2022 with a minimum follow-up time of 2 years postoperation, who had isolated *Corynebacterium* spp. From the institutional bone infection registry, we found 44 patients with PJI caused by *Corynebacterium* species, either as the sole cause or in combination with other microorganisms.

For each patient, we collected demographic data such as age and gender, data regarding the initial surgical intervention and following PJI surgical treatments, choice of antibiotic, and durations of antibiotic courses. Definite diagnoses of PJI were checked for consistency with European Bone and Joint Infection Society criteria [15]. For each patient, synovial fluid was examined for cell count and differential. During the surgery, at least 5 microbiological samples were obtained, removed material was sonicated, and multiple histopathological samples were obtained. Diagnosis of infection caused by *Corynebacterium* spp. was confirmed when at least 2 separate tissue samples or a tissue sample and a sonication sample demonstrated phenotypically identical microorganisms with matching antibiotic susceptibility profiles [15]. Each PJI was subclassified based on the timing of symptom onset after the previous surgery into postoperative, acute hematogenous, and chronic infection [16]. Additionally, infections were categorized as monomicrobial if *Corynebacterium* spp. were the sole microorganism cultured (1 or more species) and as polymicrobial if it was not the sole culprit. All patients underwent revision surgery, with the choice of surgical treatment determined individually for each case by a surgical team, following current guidelines for PJI and the institutional treatment algorithm [13, 17]. Treatment of choice was either debridement surgery with implant retention (DAIR) for early PJI (up to 4 weeks since the beginning of symptoms) and late-acute PJI (up to 3 weeks since the begining of symptoms) or implant exchange surgery for chronic PJI. Exchange surgery was either 1-stage exchange (OSE) or 2-stage exchange (TSE), depending primarily on the microbe antibiotic susceptibility, history of previous surgeries, and presence of sinus tract at the time of surgery. OSE was the choice where the microbe was known from preoperative diagnostics, no sinus tract was present, and radical joint debridability was possible. Otherwise, the TSE protocol was used for chronic PJI with an unknown microbe or a resistent microbe from preoperative diagnostics, with the sinus tract present [13, 16]. All patients were assessed using the same PJI definition, diagnostic protocols, and follow-up procedures. All patients were clinically assessed at 3 months, 6 months, 1 year, and 2 years postoperation. Treatment was considered successful if there was no relapse of PJI or death due to *Corynebacterium* spp. after 2 years of follow-up.

### Ethical Statement

This study was approved by the institutional ethical committee 5–2024 and was conducted in accordance with Declaration of Helsinki and its later amendments or comparable ethical standards.

### Statistical Analysis

For the presentation of continuous variables, due to the abnormal distribution, we used the median and interquartile range (IQR). For the presentation of descriptive variables, we used frequencies and relative frequencies. Categorical variables were analyzed using the Fisher exact test, while continuous variables were compared with the Mann-Whitney *U* test. Statistical significance was defined using a 2-sided *P* value of <.05. All statistical analyses were performed using SPSS software (version 29.0.0 for Windows; IBM Corp, Armonk, NY, USA).

## RESULTS

### Clinical Characteristics of the Cohort

Between January 2007 and December 2022, we treated 44 cases of PJI with *Corynebacterium* spp. Among them, 14 (32%) cases were monomicrobial and 30 (68%) cases were polymicrobial PJI. The demographic data of the entire *Corynebacterium* spp. cohort are shown in Table 1. The median age of patients was similar in the monomicrobial and polymicrobial groups. The time interval from the primary to the revision surgery was slightly shorter in the polymicrobial group. In both groups, the most affected joint was the hip, followed by the knee joint. In 25 (57%) cases, a revision surgery was already performed before the index revision surgery with the *Corynebacterium* spp. PJI diagnosis.

**Table 1.** Cohort Demographics for 44 PJI Cases

	All Cases (n = 44)	Monomicrobial (n = 14)	Polymicrobial (n = 30)	*P* Value
Female sex, No. (%)	24 (55)	8 (57)	16 (53)	.813
Alive at time of analysis, No. (%)	37 (84)	10 (71)	27 (90)	.242
Median age^a^ (IQR), y	73 (62–80)	74 (59–80)	73 (63–80)	.162
Median age at primary arthroplasty (IQR), y	64 (53–68)	66 (58–77)	63 (54–64)	.202
Median age at revision surgery (IQR), y	67 (56–73)	70 (61–80)	64 (55–71)	.151
Affected joint, No. (%)				
Hip	22 (50)	8 (57)	14 (47)	.747
Knee	17 (39)	4 (29)	13 (43)	.509
Ankle	2 (5)	0	2 (7)	1.00
Elbow	1 (2)	1 (7)	0	.318
Shoulder	1 (2)	0	1 (3)	1.00
Foot	1 (2)	1 (7)	0	.318
Previous surgery, No. (%)				.612
Primary revision	18 (41)	6 (14)	12 (27)	
1 previous revision	5 (11)	2 (5)	3 (7)	
2 or more previous revisions	21 (48)	5 (11)	16 (36)	

Abbreviations: IQR, interquartile range; PJI, periprosthetic joint infection.

^a^Only includes patients alive at the time of analysis.

### Infection Characteristics and Microbiological Findings

Most of the cases were classified as chronic (68%). Infection characteristics are summarized in Table 2. *Corynebacterium* strains that were isolated are listed in Table 2. Considering the microbiological isolates in our cohort, *C. striatum* was the most common microbiological isolate in monomicrobial as well as polymicrobial groups, followed by undefined *Corynebacterium* spp. strains and *C. tuberculostearicum* in the polymicrobial group. *C. tuberculostearicum* was observed in only 23% cases of polymicrobial infections; it was not observed in the monomicrobial group. Common co-pathogens in the polymicrobial group are summarized in Table 2.

**Table 2.** PJI Timing and Microbiological Findings in 44 Cases

	All Cases (n = 44)	Monomicrobial (n = 14)	Polymicrobial (n = 30)	*P* Value
Infection type, No. (%)				
Early postoperative	8 (18)	2 (14)	6 (20)	1.00
Acute hematogenous	6 (14)	2 (14)	4 (13)	1.00
Chronic	30 (68)	10 (71)	20 (67)	1.00
Median PJI revision time after last surgery (IQR), mo	22 (6–55)	26 (12–53)	18 (5–53)	.457
*Corynebacterium* spp., No. (%)				
*C. striatum*	15 (39)	7 (50)	10 (33)	.334
*C. tuberculostearicum*	7 (16)	0	7 (23)	.078
*C. jeikeium*	5 (11)	1 (7)	4 (13)	1.00
*C. amycolatum*	3 (7)	1 (7)	2 (7)	1.00
*C. accolens*	1 (2)	0	1 (3)	1.00
*C. minutissimum*	1 (2)	0	1 (3)	1.00
*C. aurimucosum*	1 (2)	0	1 (3)	1.00
Unspeciated species	15 (34)	6 (43)	9 (30)	.501
Other co-pathogens, No. (%)				
Gram-positive cocci				
Coagulase-negative staphylococci^a^			19 (63)	
*S. aureus*			6 (20)	
*Enterococcus* spp.			5 (17)	
*Streptococcus* spp.^b^			2 (7)	
Gram-negative bacilli^c^			9 (30)	
Anaerobes^d^			3 (10)	
*C. albicans*			1 (3)	

Abbreviations: CNS, central nervous system; IQR, interquartile range; PJI, periprosthetic joint infection.

^a^ *S. epidermidis* (13 cases), of which 7 were methicillin-resistant, *S. haemolyticus* (4 cases), *S. lugdunensis* (1), *S. hominis* (1), and 2 unspeciated CNS strains.

^b^ *S. agalactiae* (1) and *S. gallolyticus* (1).

^c^ *P. aeruginosa* (3), *E. cloacae* (1), *S. maltophilia* (1), *E. coli* (1), *P. mirabilis* (1), *A. baumannii* (1), *K. pneumoniae* (1).

^d^ *F. magna* (1), *P. harei* (1), and *C. acnes* (1).

### Management

Surgical and antimicrobial treatment is summarized in Table 3. The DAIR procedure was performed in 14 cases (32%), where the mean duration of symptoms was 10 days, with a maximum of 30 days. Implant exchange was conducted in 30 cases (68%), all >3 months after the previous surgery, with 1 revision performed 3 days after the first surgery. In the implant exchange group, 7 cases were treated with the OSE from them 5 monomicrobial, and 23 with the TSE from them 5 monomicrobial.

**Table 3.** Surgical and Antimicrobial Management of 44 PJI Cases

	All Cases (n = 44)	Monomicrobial (n = 14)	Polymicrobial (n = 30)	*P* Value
Surgical management, No. (%)				
DAIR	14 (32)	4 (29)	10 (33)	1.00
Implant exchange	30 (68)	10 (71)	20 (67)	1.00
OSE	7 (16)	5 (36)	2 (7)	.025
TSE	23 (52)	5 (36)	18 (60)	.197
Antimicrobial treatment p.o.,^a^ No. (%)				
Minocycline	13 (30)	4 (29)	9 (30)	1.00
Doxycycline	2 (5)	1 (7)	1 (3)	.540
Amoxicillin	10 (23)	4 (29)	6 (20)	.701
Co-amoxiclav	3 (7)	1 (7)	2 (7)	1.00
Ciprofloxacin	9 (20)	2 (14)	7 (23)	.659
Clindamycin	6 (14)	1 (7)	5 (17)	.647
Co-trimoxazole	2 (5)	1 (7)	1 (3)	.540
Fusidic acid	1 (2)	0 (0)	1 (3)	1.00
No oral treatment	4 (9)	1 (7)	3 (10)	1.00
>1 antimicrobial agent p.o., No. (%)	22 (50)	7 (50)	15 (50)	1.00
Antimicrobial treatment, only i.v., No. (%)	3 (7)		3 (10)	/
Rifampicin addition, p.o.,^a^ No. (%)	13 (30)	4 (29)	9 (30)	1.00
Median duration of p.o. antimicrobial treatment (IQR), d	84 (84–84)	84 (78–84)	84 (84–84)	.205
P.o. antimicrobial treatment duration >84 d^a^	4 (9)	0	4 (13)	.290

Abbreviations: DAIR, debridement, antibiotics, and implant retention; IQR, interquartile range; OSE, 1-stage exchange; p.o., per os (oral); PJI, periprosthetic joint infection; TSE, 2-stage exchange.

^a^Forty out of 44 patients received p.o. antimicrobial treatment.

In general, all patients received 2 g of cefazolin 30 minutes before skin incision. The patients colonized with MRSA or allergy to penicillin received 2 g of vancomycin 2 hours before skin incision. After surgery, the patients without a sinus tract received amoxicillin-clavulanic acid 1.2 g/8 hours and vancomycin (in general, an initial loading dose of 2 g followed by dosing guided by measurement of the vancomycin serum concentration), and the patients with a sinus tract present received piperacillin-tazobactam 4.5 g/8 hours and vancomycin. In the case of septic patients, imipenem-cilastatin 500 mg/6 hours and vancomycin were used. As soon as the microbiological results of the antimicrobial testing of the insulated bacteria were obtained, antibiotic treatment was downgraded according to the susceptibility profile of the insulated bacteria. In general, piperacillin-tazobactam or imipenem-cilastatin was stopped 2–5 days postoperatively. All patients received an initial 10–14-day intravenous course of antibiotic treatment, which was followed by oral antimicrobials in 41 (90%) patients, with a median duration of 84 days in both the monomicrobial and polymicrobial groups. The anaerobic microbiological cultures were left cultivated for 14 days for a definite result. In 3 cases (10%) from the polymicrobial group, antimicrobial treatment was applied exclusively intravenously. In these patients there was no oral antibiotic option. For all these patients the 2-stage protocol was used. The duration of intravenous therapy in those cases was 6 weeks, as is usual in osteomyelitis cases. Longer antimicrobial treatment was needed in the polymicrobial group in 4 cases (13%). Additionally, all these patients had isolated *Streptococcus* spp., where 1 year of oral antibiotic treatment was prescribed according to hospital protocol based on the findings of Renz et al. on long-term antimicrobial suppression in streptococcal periprosthetic joint infections [18]. The oral antimicrobial agents are summarized in Table 3; the most-used antibiotic was minocycline, used in 13 cases (30%). Rifampicin was added in 13 cases (30%), and 50% of patients received a combination of ≥2 antimicrobial agents. Rifampicin itself was used only as an oral treatment. In general, it was introduced to therapy when drains were removed and no secretion from the wound was noted by 5–7 days postsurgery. Dosing of rifampicn was 450 mg/12 hours.

Oral antibiotics were used depending on the susceptibility of the strains in the monomicrobial group, most commonly minocycline and amoxicillin, while in the polymicrobial group ciprofloxacin and clindamycin were used in addition to minocycline and amoxicillin. The most common antibiotic combinations were ciprofloxacin-rifampicin and minocycline-rifampicin, each in 5 (11%) cases. Antibiotic combinations are presented in Table 4.

**Table 4.** Antibiotic Combinations in 44 PJI Cases

	All Cases (n = 44)	Monomicrobial (n = 14)	Polymicrobial (n = 30)
Antibiotic combinations, No. (%)			
Amoxicilin	6 (14)	3 (21)	3 (10)
Amoxicilin-ciprofloxacin	2 (5)	1 (7)	1 (3)
Amoksicilin-minocycline	2 (5)	0	2 (7)
Co-amoxiclav	3 (7)	1 (7)	2 (7)
Ciprofloxacin	1 (2)	0	1 (3)
Ciprofloxacine-minocycline	1 (2)	0	1 (3)
Ciprofloxacin-rifampicin	5 (11)	1 (7)	4 (13)
Doxycycline	1 (2)	0	1 (3)
Doxycycline-rifampicin	1 (2)	1 (7)	0
Fusidic acid–rifampicin	1 (2)	0	1 (3)
Minocycline	4 (9)	2 (14)	2 (7)
Minocycline-rifampicin	5 (11)	2 (14)	3 (10)
Minocycline-co-trimoxazole-rifampicin	1 (2)	0	1 (3)
Clindamycin	6 (14)	1 (7)	5 (17)
Co-trimoxazole	1 (2)	1 (7)	0
No oral treatment	4 (9)	1 (7)	3 (10)

Abbreviation: PJI, periprosthetic joint infection.

In the monomicrobial group, 10 (71%) patients were treated with reimplantation and in 4 (29%) with DAIR. An antibiotic combination including rifampicin was used in 4 cases (29%), of which 2 were DAIR and 2 were reimplantation. Similarly, in the polymicrobial group, 20 (67%) were treated with reimplantation and 10 (33%) with DAIR. In 9 (30%) cases, rifampicin was used, 5 of which were DAIR and 4 of which were reimplantation.

### Treatment Outcome

For our cohort, with well-established DAIR suitability criteria, we observed similar treatment success rates of DAIR and implant exchange, 93% and 90% respectively, at 2-year follow-up. Of all PJI cases 40 were successfully treated after the first revision, followed by a course of antimicrobial treatment. Additionally, 4 cases from the polymicrobial group required a second revision procedure, all of which were successful. Within the polymicrobial group, DAIR was successful in 90% (9/10) of cases, while reimplantation was successful in 85% (17/20). Further details on outcomes are provided in Table 5.

**Table 5.** Treatment Outcome in 44 PJI Cases

	All Cases (n = 44)	Monomicrobial (n = 14)	Polymicrobial (n = 30)	*P* Value
First revision success, No. (%)				
Successful	40 (91)	14 (100)	26 (87)	.290
Second revison required	4 (9)	0	4 (13)	.290
Successful after second revision	4 (9)		4 (13)	
DAIR, No. (%)	n = 14	n = 4	n = 10	
Combination with rifampicin	7 (50)	2 (50)	5 (50)	1.00
Successful outcome	13 (93)	4 (100)	9 (90)	1.00
Implant exchange, No. (%)	n = 30	n = 10	n = 20	
Combination with rifampicin	6 (20)	2 (20)	4 (20)	1.00
Successful outcome	27 (90)	10 (100)	17 (85)	.532
First revision success considering surgical treatment option used, No. (%)	n = 40			1.00
DAIR	13 (93)			
OSE	7 (100)			
TSE	20 (87)			

Abbreviations: DAIR, debridement, antibiotics, and implant retention; OSE, 1-stage exchange; PJI, periprosthetic joint infection; TSE, 2-stage exchange.

## DISCUSSION

The observed prevalence of CPJI is 6% (44 out of 750) for our institution. Similar to previous studies on CPJI, we found a low prevalence of those infections, especially monomicrobial PJI. Our overall success rate was 91%, which is higher than in most other studies, reporting success rates between 50% and 85% [2–4, 19–22]. Probable reasons for the difference in observed success rate could be the systematic use of rifampicin in our cohort which has already been described to have an important role in treating infections with staphylococcal biofilms, while the role of rifampicin in corynebacterial infections is still controversial [23]. There is currently no consensus on the most appropriate antimicrobial treatment regimen in cases of CPJI. The course of antibiotics is mostly chosen according to the species’ susceptibility to various drugs. Some studies report that vancomycin, linezolid, and daptomycin are the most appropriate choices, with up to 100% susceptibility [2, 3, 19–22, 24]. With *Corynebacterium* spp., the problem of multidrug resistance is important, especially resistance to aminoglycosides, penicillin and cephalosporines, in up to 60% of isolated strains [21]. In our study, our use of antibiotics depended on the susceptibility of strains in the monomicrobial group, most commonly minocycline and amoxicillin, while in the polymicrobial group ciprofloxacin and clindamycin were used in addition to minocycline and amoxicillin. Our selection of antibiotics differs from most studies [2–4, 19, 21, 22], to which we can probably contribute the success of our outcomes. Two other studies mention the use of amoxicillin as a drug of choice in cases of susceptibility [20, 25], while only one study available advocates for the use of rifampicin if susceptibility is appropriate [25]. In our case, we used rifampicin in about a third of all cases.

In our cohort, the treatment success rate was 100% in the monomicrobial group and 85% in the polymicrobial group. Among 4 cases of treatment failure in the polymicrobial group, 1 case was treated with the DAIR protocol and 3 cases with implant exchange. It is important to consider that most available studies lack clear division of polymicrobial and monomicrobial infections, which is why comparison of treatment success with other studies is difficult to establish for these 2 groups [3, 4, 19–22]. The only study available concentrating on monomicrobial infections reports success rates of 71% and 89% for DAIR and TSE, respectively [2]. Similar studies including polymicrobial infections report lower treatment success, with treatment failure approaching up to 40% [3, 20]. Additionally, several studies report on the importance of proper choice of surgical treatment in corynebacterial infections, reporting that DAIR is less successful than implant exchange [2, 20, 22, 24]. In our cohort, with well-established DAIR suitability criteria, we observed similar success rates of DAIR and implant exchange, 93% and 90%, respectively.

Considering the microbiological isolates isolated in our cohort, *C. striatum* was the most common microbiological isolate in monomicrobial as well as polymicrobial infections, followed by undefined *Corynebacterium* spp. strains and *C. tuberculostearicum* in the polymicrobial group. Polymicrobial infections including *Corynebacterium* spp. have been reported to be more common than monomicrobial, which was true in our cohort as well [10]. In our cohort, we observed no evidence for certain *Corynebacterium* spp. being associated with higher failure rates, while some studies have reported that *C. striatum* is the most common species but have observed less success in treating PJI [2, 3, 20, 22, 24]. Some studies on corynebacterial bone and joint infections have reported that *C. tuberculostearicum* is the most common pathogen, while we observed this isolate in only 23% of the polymicrobial PJIs [19, 20].

As an important limitation of our study, we have to emphasize the retrospective analysis of prospectively collected data. Additionally, due to low prevalence of PJIs with isolated *Corynebacterium,* the period of patient inclusion in our study was quite long.

## CONCLUSIONS

In contrast with the previously published papers on CPJI, we observed a good microbiological cure rate following the established algorithm, with a total cure rate of 91% for our cohort. As expected, the cure rate was slightly lower in the polymicrobial group compared with the monomicrobial, at 87% and 100%, respectively.
